# The Role of Upregulated *DDX11* as A Potential Prognostic and Diagnostic Biomarker in Lung Adenocarcinoma

**DOI:** 10.7150/jca.33457

**Published:** 2019-07-10

**Authors:** Jianhao Li, Liwen Liu, Xin Liu, Penglin Xu, Qiuyue Hu, Yan Yu

**Affiliations:** 1 Precision Medicine Center, The First Affiliated Hospital of Zhengzhou University, Zhengzhou 450052, China; 2 Key Laboratory of Clinical Medicine, The First Affiliated Hospital of Zhengzhou University, Zhengzhou 450052, China

**Keywords:** *DDX11*, lung adenocarcinoma, diagnosis, proliferation, cell cycle

## Abstract

**Background**: Lung adenocarcinoma (ADC) is the main cause of cancer-related mortality in lung cancer patients. DEAD/DEAH box helicase 11 (*DDX11*) was previously shown to be dysregulated and to exert oncogenic activity in cancer. However, the diagnostic value and clinical significance of *DDX11* in ADC remain unknown.

**Methods**: A total of 513 ADC and 59 normal tissue samples were obtained from The Cancer Genome Atlas (TCGA) database, and the mRNA expression level of *DDX11* in ADC was evaluated. Additionally, a meta-analysis of 7 ADC cohorts from the Gene Expression Omnibus (GEO) database was conducted to validate the *DDX11* expression pattern. Moreover, receiver operating characteristic (ROC) curve analysis was used to identify the diagnostic power of *DDX11* in ADC. A tissue microarray (TMA) comprising 86 ADC specimens and their adjacent normal specimens was applied to indicate *DDX11* protein expression status. In addition, Kaplan-Meier and Cox regression analyses were conducted to validate the prognostic value of *DDX11* in ADC. Finally, the molecular mechanism of *DDX11* action in ADC was predicted by gene set enrichment analysis (GSEA).

**Results**: *DDX11* was upregulated in ADC tissues and was associated with worse overall survival (OS). ROC curves of *DDX11* showed high values for diagnosis. Additionally, *DDX11* expression* has* remarkable correlations with DNA replication and the cell cycle G1-S phase pathway. Consistently, it was associated with cell cycle genes, such as *CCNA2*, *CCNB1*, *CCNC*, *CCND1*, *CCNE1*, *CDK2*, *CDK4* and *CDK6*. Moreover, high *CCNA2*, *CCNB1*, *CCNE1* and *CDK6* expression in ADC patients predicted worse OS and progression-free survival (PFS).

**Conclusion**: *DDX11* was significantly upregulated and predicted poor prognosis in ADC. This gene might serve as a potential novel prognostic and diagnostic biomarker for ADC.

## Introduction

Lung cancer is one of the main causes of cancer-associated mortality worldwide. This disease is subdivided into non-small cell lung cancer (NSCLC) and small cell lung cancer, with NSCLC accounting for approximately 80-85% of all lung cancers 1, 2. Additionally, lung adenocarcinoma (ADC) is the most frequent subtype of NSCLC, accounting for approximately 40% of these cancers 3. Although great advances have been made in the diagnosis and treatment of ADC, such as chemotherapy, immunotherapy and targeted therapy 4, 5, the 5-year overall survival (OS) rate of ADC patients with advanced stage is approximately 50%-70% due to the malignant features of high metastasis and recurrence 6, 7. Therefore, powerful diagnostic and therapeutic strategies are still urgently needed to improve the prognosis of ADC patients.

The *DDX11* (alias *ChlR1*) gene is located on human chromosome 12p11 and encodes an orthologue of the yeast gene *Chl1*, which is a member of the DEAD/DEAH box family of ATP-dependent helicases 8, 9. *DDX11* plays a significant role in the cohesion of chromosome arms and centromeres. Mitotic failure occurs due to replicated chromosomes failing to segregate after prometaphase arrest when *DDX11* is depleted 10, 11. Previous studies have proven that *DDX11* biallelic mutations cause Warsaw breakage syndrome 12-14. Additionally, a previous study suggested that the *DDX11* expression level is high in melanomas and plays a key role in cancer progression 15. However, until now, there have been no relevant studies focused on the expression and function of *DDX11* in ADC.

The findings of our study indicated that the *DDX11* expression level was significantly higher in ADC tissues than in adjacent normal tissues. Then, we observed that high *DDX11* expression was associated with poor prognosis. Further, receiver operating characteristic (ROC) curve and meta-analysis showed a reliable diagnostic value for *DDX11* in ADC patients. The expression of *DDX11* was crucially correlated with the cell cycle G1-S phase and the DNA replication pathway. In summary, our results indicated that *DDX11* might be a promising diagnostic and prognostic biomarker for ADC.

## Materials and Methods

### TCGA data source

The data for 513 ADC and 59 normal tissue samples from The Cancer Genome Atlas (TCGA, https://tcga-data.nci.nih.gov/tcga/) database were downloaded for gene expression analyses and survival analyses. A total of 500 of the 513 ADC patients with follow-up survival time information were divided into higher and lower *DDX11* expression groups by using X-tile, a recently developed tool for the evaluation of biological relevance between a biomarker and the patient outcome, and the discovery of population cut-points based on marker expression 16. The survival analysis was conducted using the Kaplan-Meier method and a log-rank test. The raw data were analysed by BRB-array tools as previously reporte 17, 18.

### GEO data source

Seven ADC datasets accompanied with scientific publications (GSE27262, GSE30219, GSE31210, GSE33532, GSE30219, GSE7670, and GSE10072) were gathered through the GEO database (http://www.ncbi.nlm.nih.gov/geo/, Gene Expression Omnibus). Then, we used meta-analysis to evaluate the diagnostic value of *DDX11*. The characteristics of the datasets, such as Cohort ID, RNAseq platforms, number of samples (tumour and non-tumour samples), publication year and country, are presented in Table S1.

### Tissue samples

A microarray of 86 ADC tumour and adjacent normal tissue samples, which was constructed utilizing a core diameter of 1.5 mm, was obtained from a commercial tissue microarray analysis (TMA) company (Shanghai OutdoBiotech, China). All experiments were approved by the Ethics Committee of the First Affiliated Hospital of Zhengzhou University, Zhengzhou, China.

### GSEA

GSEA was used to confirm the distribution of the individual genes of TCGA ADC datasets. The expression profiles of 513 samples from TCGA database were divided into two groups according to gene expression. GSEA v2.0 was then performed to verify whether the gene sets from the MSigDB database v4.0 are positively related to the expression of *DDX11*. The statistical significance threshold was set at P < 0.05.

### Statistics for the meta-analysis

A meta-analysis was carried out to examine the pooled diagnostic power of *DDX11* with the data from the GEO database using Stata software. We assessed heterogeneity among studies using *I^2^* statistics. When *I^2^*>50%, significant heterogeneity would be considered, and a random model would be performed for the meta-analysis. The subgroup analysis was carried out according to the following factors: region, sample size and publication year. Begg's test and Egger's test were used to determine the bias of the publications.

### Immunohistochemistry (IHC)

IHC was performed as previously reported 19, 20. Briefly, 5 μm thick TMA sections were deparaffinized and then treated with hydrogen peroxide to quench endogenous peroxidase activity. Subsequently, the sections were incubated overnight with a rabbit anti-human *DDX11* antibody (1:200, Abcam, USA) at 4℃. Then, the immunoreactive cells were detected by Signal Stain® DAB (CST, USA) and counterstained with Haematoxylin QS (Vector Laboratories). Two experienced pathologists, who were blinded to the clinicopathological data, separately evaluated the immunostaining samples, and the samples were scored according to the proportion of positive cells as follows: 1, <25%; 2, 25%-50%; 3, 51%-75%; and 4, 76%-100%. The staining intensity was scored as follows: 0, no staining; 1+, weak staining; 2+, moderate staining; 3+, strong staining; and 4+, and intense staining. The multiply of the two sub scores (range 0-16) was classified low expression (range 0-8) and high expression (range 8-16), respectively for statistical analysis.

### Statistical analysis

All statistical analyses were performed using GraphPad Prism software (version 7.0, GraphPad Software, Inc., La Jolla, CA, USA) and SPSS software (version 23.0, SPSS Inc., Chicago, IL). A chi-squared test was used to examine the correlation between *DDX11* expression levels and the clinicopathological parameters. Kaplan-Meier curves were utilized to analyse the OS of ADC patients. Cox regression analysis of univariate and multivariate variables was used to indicate the relationship between the different variables and survival. ROC curves were utilized to examine the pooled diagnostic power of *DDX11* in ADC. Heatmaps were used to show the patterns of mRNA expression according to tumour-node-metastasis (TNM) and histological type. Pearson's correlation was performed to ascertain the linear correlation between 2 variables. P<0.05 is regarded as statistically significant. All data are presented as the means ± SD. All experiments were carried out at least three times.

## Results

### *DDX11* mRNA is upregulated and correlated with poor prognosis in ADC

**Figure 1:**
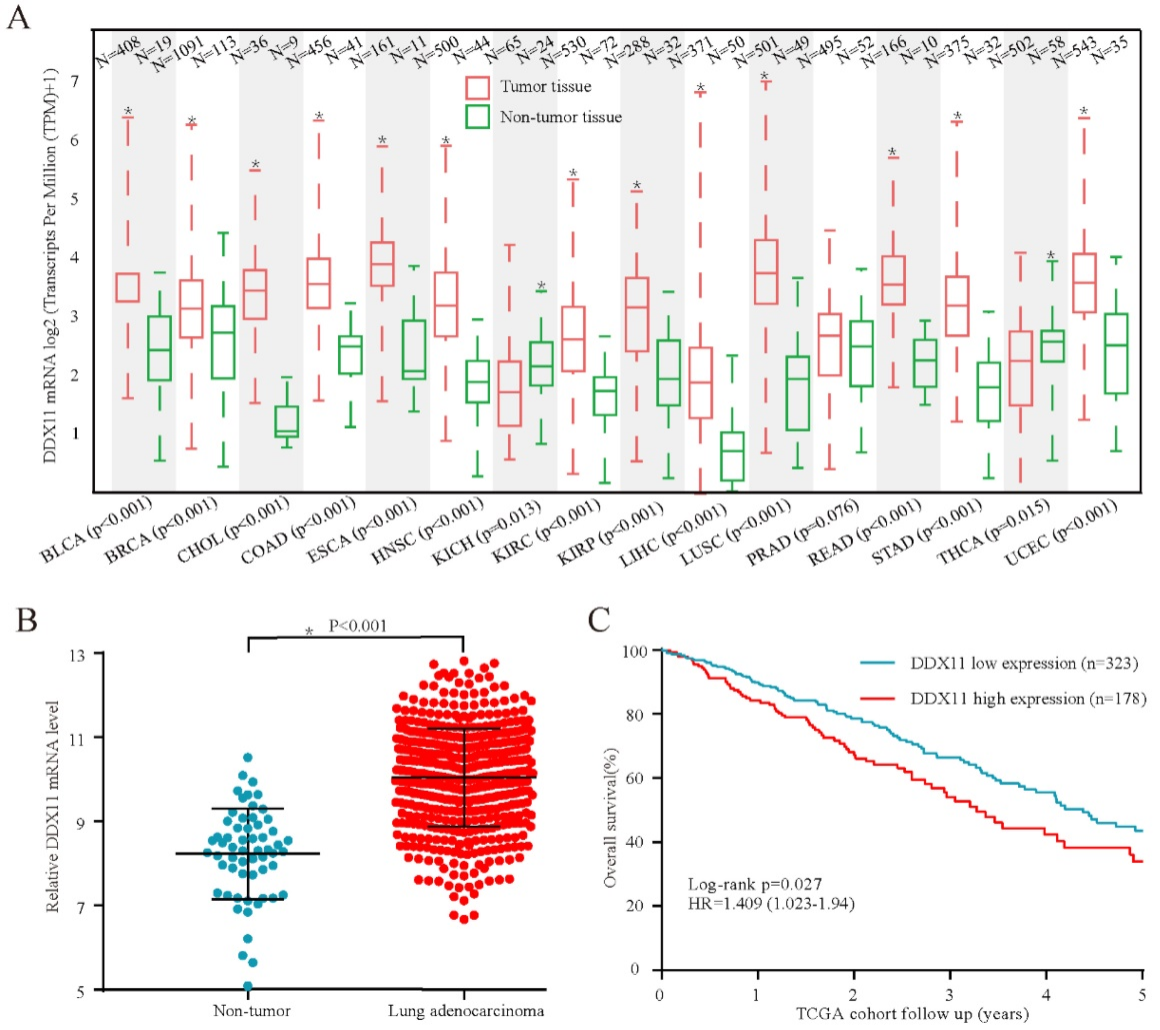
***DDX11* mRNA was overexpressed in ADC tissues and negatively correlated with survival in TCGA cohort. Notes: (A and B)** *DDX11* mRNA expression in non-tumour tissues and ADC tumour tissues. **(C)** Kaplan-Meier estimation of the OS of ADC patients stratified by *DDX11* expression. **Abbreviations:** *DDX11*: DEAD/DEAH box helicase 11; OS: overall survival; ADC: adenocarcinoma.

To investigate the expression of *DDX11* in cancers, TCGA data analysis was conducted to identify *DDX11* mRNA expression levels. The results demonstrated that *DDX11* mRNA was highly expressed in numerous tumour samples compared with its expression in non-tumour tissues (Figure 1A). Consistently, *DDX11* is upregulated in tumour tissues of ADC (Figure 1B). Moreover, survival analysis revealed that the high expression of *DDX11* could predict poorer overall survival (OS) (Figure 1C). Taken together, these results indicate that *DDX11* might be a novel prognostic biomarker for ADC patients.

### Upregulated *DDX11* protein is associated with clinicopathological characteristics and the poor prognosis of ADC

**Figure 2:**
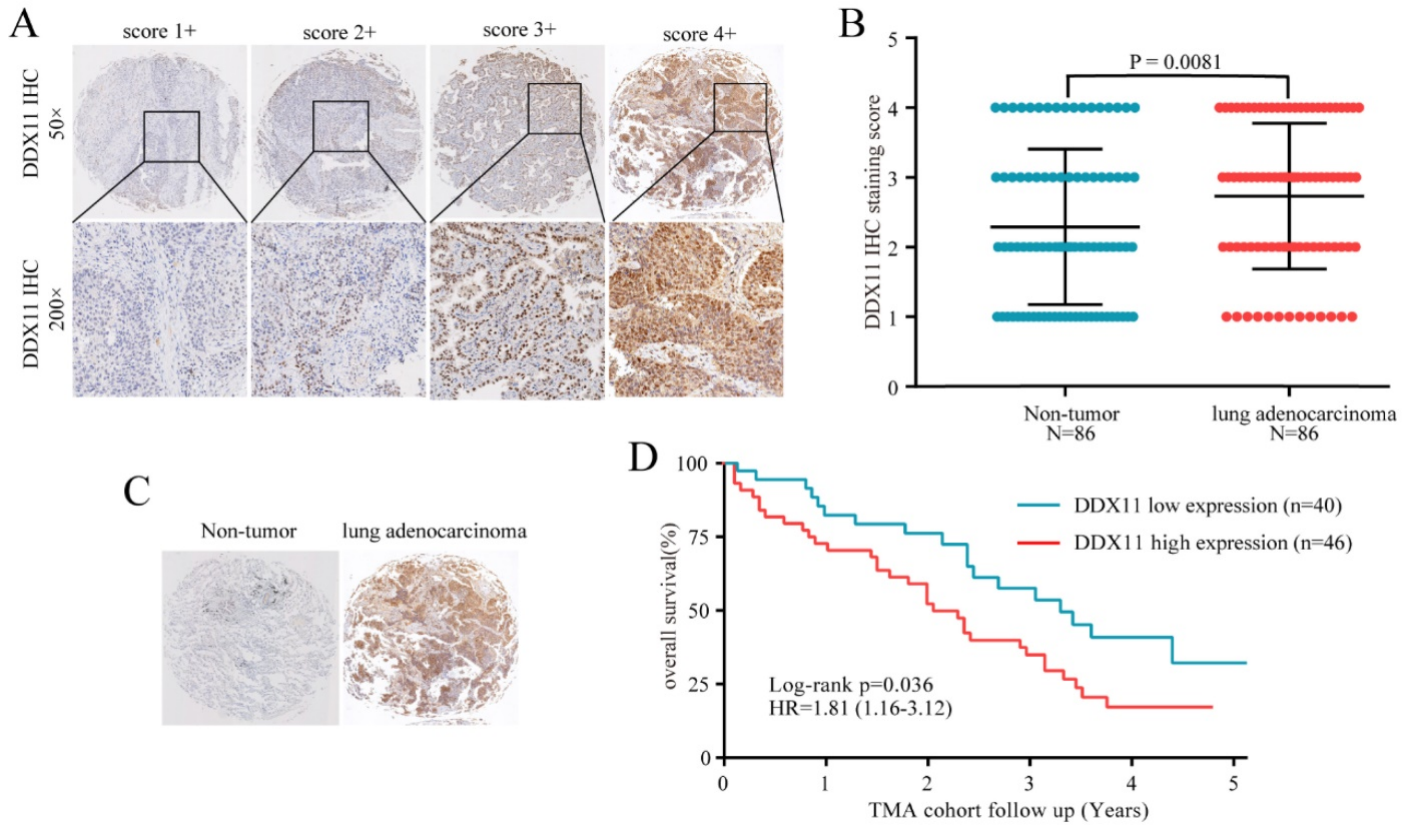
**High expression of *DDX11* protein was negatively correlated with survival. Notes: (A)** Representative images of *DDX11* staining in ADC tissues. **(B)** Increased expression of *DDX11* in ADC tissues (P=0.0081). **(C)** Representative *DDX11* staining in ADC and non-tumour tissues. **(D)** Kaplan-Meier analysis showing the correlation between *DDX11* expression levels and the OS of 86 ADC patients. **Abbreviations:** *DDX11*: DEAD/DEAH box helicase 11; ADC: lung adenocarcinoma; IHC: immunohistochemistry; OS: overall survival.

Subsequently, considering the expression difference in mRNA, we performed immunohistochemistry to evaluate the *DDX11* protein expression status in ADC. According to the staining intensity, *DDX11* staining was scored from 1+ to 4+ (Figure 2A). A score of 1+ to 2+ was defined as low *DDX11* expression, whereas a score of 3+ to 4+ was defined as high *DDX11* expression. Consistent with the results of TCGA and GEO database analyses, the expression levels of *DDX11* protein were significantly upregulated in ADC tissues (Figure 2B). Furthermore, *DDX11* expression was significantly positively related to tumour size and the TNM stage of the patients (Table 1). Additionally, Kaplan-Meier analysis indicated that high expression of *DDX11* was remarkably correlated with poor OS in ADC patients (P = 0.036, Figure 2D). Moreover, univariate and multivariate analyses demonstrated that, in addition to the TNM stage, *DDX11* might be an independent prognostic factor for ADC patients (Table 2). In summary, these findings strongly suggested that* DDX11* might serve as a prognostic biomarker in ADC.

### A meta-analysis on the diagnostic value of *DDX11* in ADC through the GEO database

**Figure 3:**
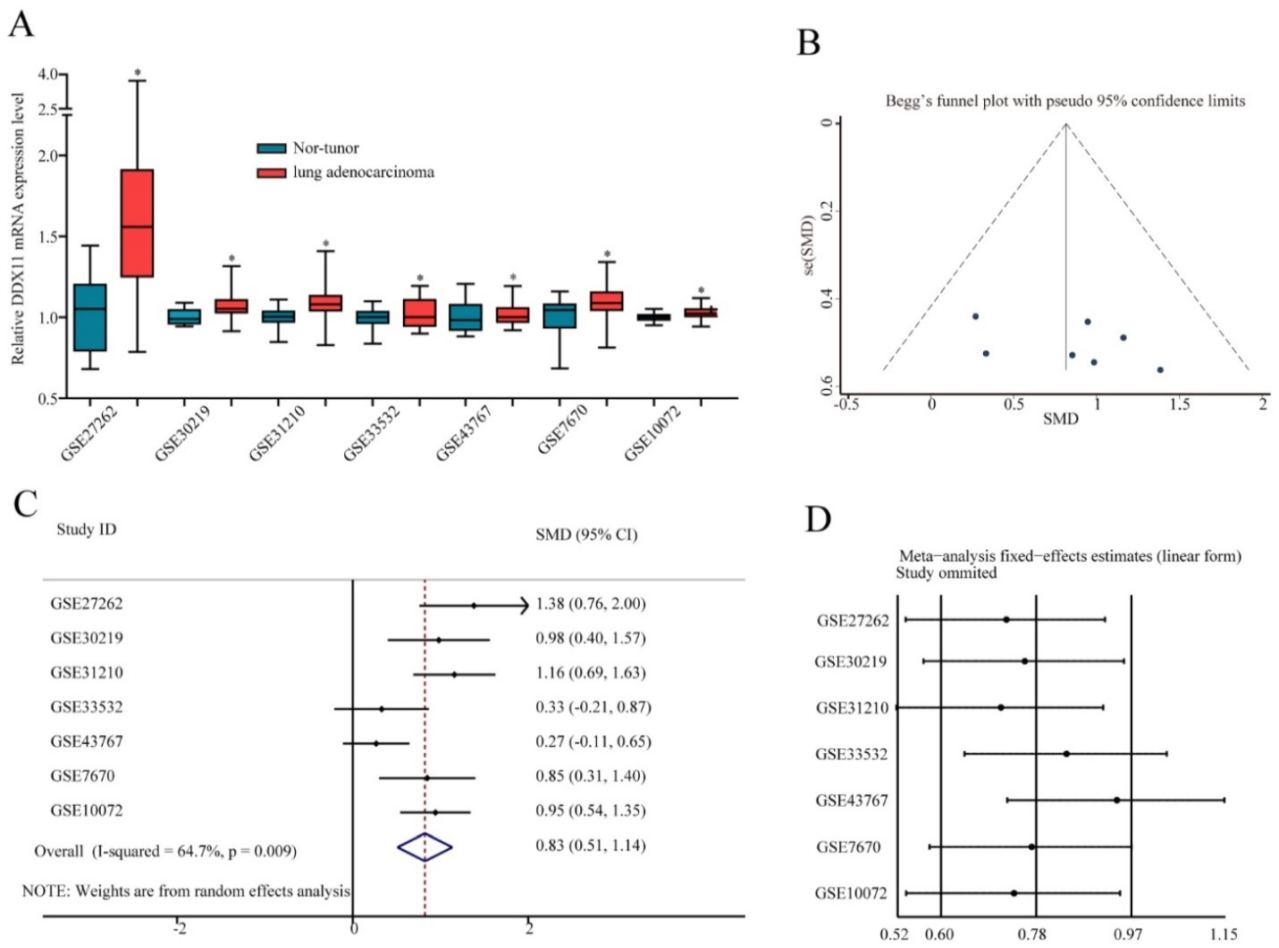
***DDX11* expression was markedly increased in ADC tissues and showed high diagnostic value in the GEO dataset. Notes: (A)** *DDX11* expression in ADC and normal tissues. **(B)** Expression between ADC and normal tissues. **(C)** Forest plot evaluating differences in *DDX11* expression between ADC and normal tissues. The high and low *DDX11* expressing tissues were regarded as the experimental and control groups, respectively. **(D)** Sensitivity analysis of the hazard ratios was calculated by omitting each microarray in turn. **Abbreviations:** *DDX11*: DEAD/DEAH box helicase 11; GEO: Gene Expression Omnibus; ADC: adenocarcinoma; SMD: standard mean difference; CI: confidence interval.

**Figure 4:**
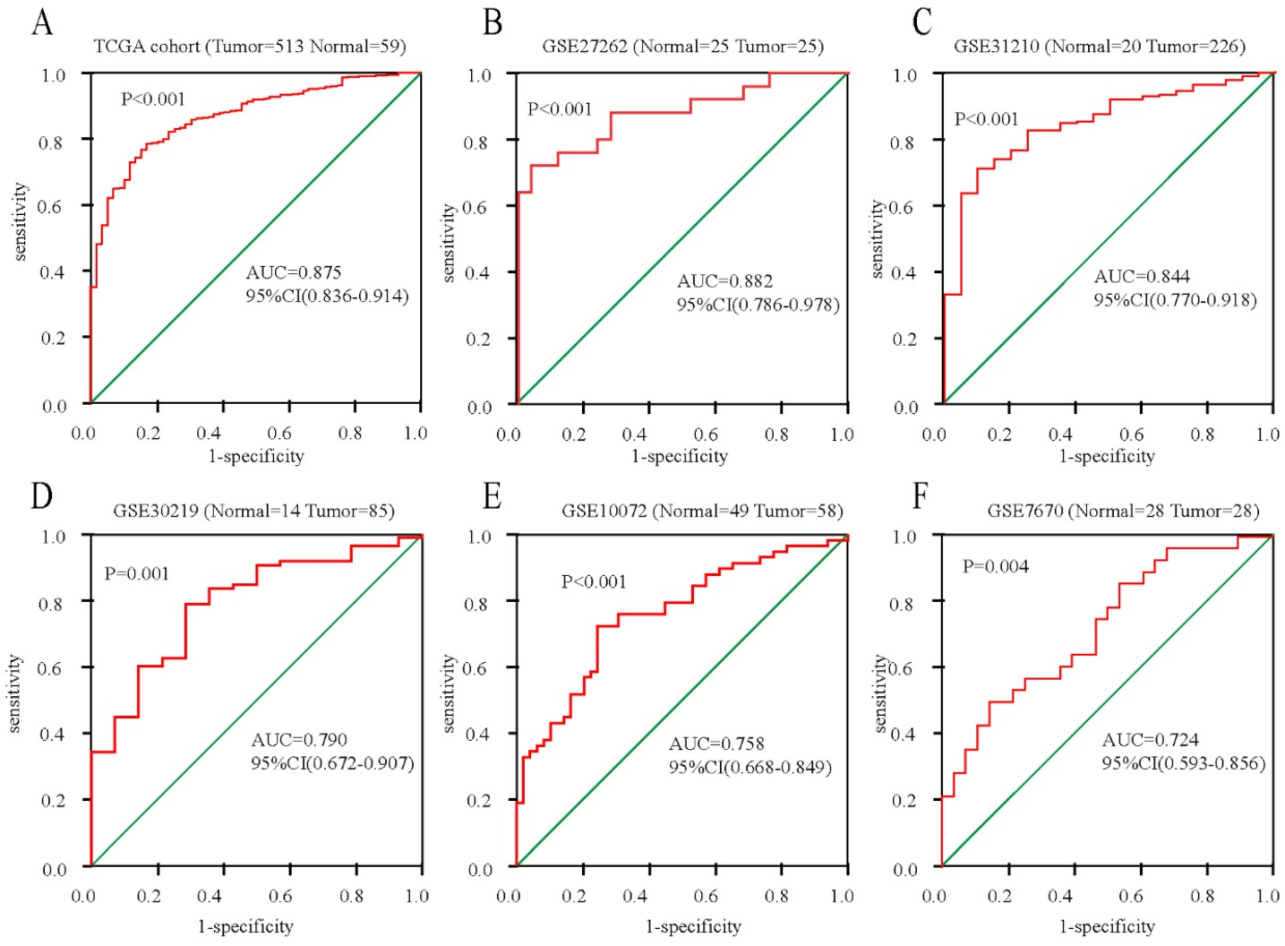
** ROC curves for evaluating the diagnostic power of *DDX11* in ADC. Notes: (A)** TCGA cohort. **(B)** GSE27262. **(C)** GSE31210. **(D)** GSE30219. **(E)** GSE10072. **(F)** GSE7670. **Abbreviations:** AUC: area under the curve; *DDX11*: DEAD/DEAH box helicase 11: ADC: adenocarcinoma; ROC: receiver operating characteristic; TCGA: The Cancer Genome Atlas; CI: confidence interval.

To further confirm the mRNA expression of *DDX11* in ADC, a total of 7 microarrays in the GEO database were collected and extracted in the present study. As shown in the forest plot (Figure 3A), because of the significant heterogeneity among the micrograms (I^2^ value was 64.7%), a random effect model analysis was performed, showing that the expression of *DDX11* was higher tumour tissue than in non-tumour tissue (pooled standard mean difference (SMD)=0.83, 95% CI=(0.51-1.14), P=0.009). The results are consistent in Figure 3C. Sensitivity analyses noted that there were no significant differences (Figure 3B and 3D). Begg's test (P =0.230) and Egger's test (P =0.288) showed no statistical significance. In total, there was no significant publication bias among these studies. In Figure 4, the ROC analysis revealed a significant diagnostic value in ADC. The results of the ROC analysis from TCGA database are shown in Figure 4A (area under the curve (AUC), 0.875; 95% CI, 0.0.836-0.914; P<0.001), and the corresponding specificity and sensibility were 0.793 and 0.831, respectively. The AUC was 0.882 (95% CI: 0.786-0.978. P < 0.001) in GSE27262, and the corresponding specificity and sensibility were 0.72 and 0.96, respectively. The specificity and sensibility were 71% and 90%, respectively. The AUC was 0.844 (95% confidence interval (CI): 0.770-0.918. P < 0.001) in GSE31210, and the corresponding specificity and sensibility were 0.79 and 0.71, respectively. The AUC of *DDX11* expression was 0.790 (95% CI: 0.672-0.907, P = 0.001) in GSE30219. The AUC was 0.758 (95% CI: 0.668-0.849. P < 0.001) in GSE10072, and the corresponding specificity and sensibility were 0.72 and 0.76, respectively. The AUC was 0.724 (95% CI: 0.593-0.856. P = 0.004) in GSE7670, and the corresponding specificity and sensibility were 0.5 and 0.857, respectively. In summary, *DDX11* could be a possible indicator to assist the diagnosis of ADC.

### The potential molecular mechanism mediated by *DDX11* in ADC

**Figure 5:**
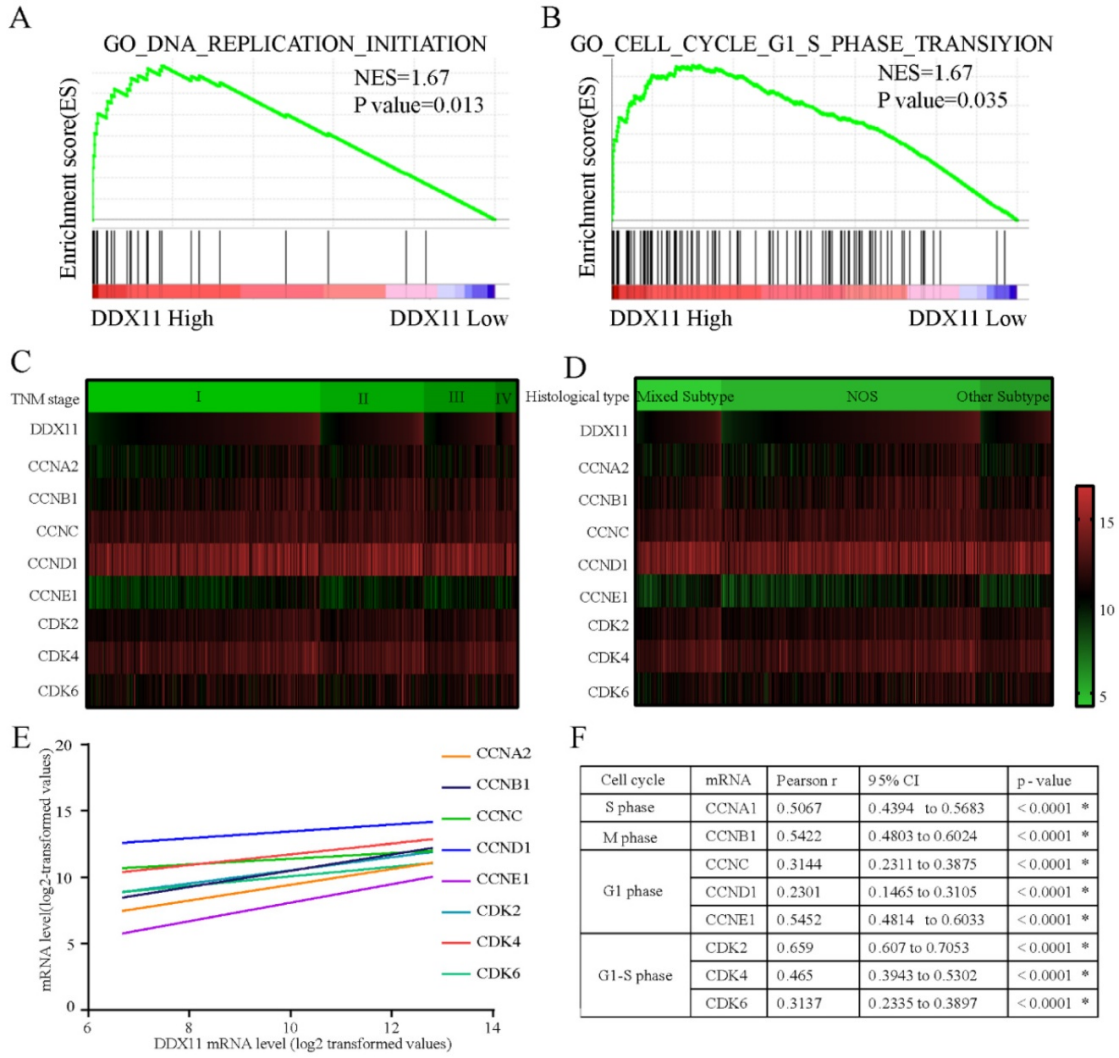
**Molecular mechanism of *DDX11* action in ADC. Notes:** (A B) GSEA of the relationship between high *DDX11* expression and genes associated with cell cycle G1_S_phase (GO _ cell _ cycle_G1_S_phase) and DNA replication (GO _ DNA_ replication). (C D) Heatmap of mRNA expression according to TNM and histological type. The genes assessed were CCNA2, CCNB1, CCNC, CCND1, CCNE1, CDK2, CDK4 and CDK6. (E F) Correlation of *DDX11* mRNA expression with the other genes. **Abbreviations:** GSEA: gene set enrichment analysis; *DDX11*: DEAD/DEAH box helicase 11; NES: normalized enrichment score; CI: confidence interval; NOS: not otherwise specified; TNM: tumor node metastasis.

To explore the underlying mechanisms by which *DDX11* is involved in ADC progression, we conducted a GSEA based on TCGA ADC cohort. The GSEA showed that *DDX11* upregulation was associated with the activation of DNA replication and the cell cycle G1-S phase transition pathway (Figure 5A and 5B). Cell cycle-related gene expression patterns according to TNM stage and histological type were described in a heatmap plot of 505 ADC patients from TCGA database (Figure 5C and 5D). Additionally, we found a significant positive relationship between the *DDX11* expression level and the genes involved in the cell cycle G1-S phase transition and DNA replication (Figure 5E and 5F), such as *CCNA2* (P < 0.0001, R=0.5067), *CCNB1* (P < 0.0001, R=0.5422), *CCNC* (P < 0.0001, R=0.3114), *CCND1* (P < 0.0001, R=0.2301), *CCNE1* (P < 0.0001, R=0.5452) and *CDK2* (P < 0.0001, R=0.659), *CDK4* (P < 0.0001, R=0.465), and *CDK6* (P < 0.0001, R=0.3137). Moreover, ADC patients with high expression of *CCNA2*, *CCNB1*, *CCND1*, *CCNE1*, and *CDK6* had a worse OS, and *CCNA2*, *CCNB1*, *CCNE1* and *CDK6* were associated with worse PFS. These findings showed that *DDX11* likely contributed to the poor prognosis of ADC through cell proliferation.

## Discussion

ADC accounts for almost 50% of lung cancers, and although the diagnostic and therapeutic techniques for ADC have made significant progress, the 5-year OS for ADC patients remains poor 21. Therefore, it is of vital importance to elucidate the molecular mechanisms of ADC development and identify novel prognostic biomarkers and therapeutic targets for ADC. A few proteins, including *HMGA1* 22, *IDH1* 23, *CEA* and *CYFRA* 24, have been reported to be differentially expressed in ADC and associated with ADC progression.

*DDX11*, a DNA-dependent ATPase and helicase, is involved in the processing of the lagging strand during DNA replication and in the maintenance of the fork structure for the establishment of cohesion 25, 26. Recent studies have shown an oncogenic function for *DDX11* in a few cancers. For example, Bhattacharya *et al.* reported that high *DDX11* expression was significantly related to poor prognosis in advanced melanomas. However, its functional role and clinical significance in ADC have never been reported. In this study, we consistently found high *DDX11* expression in ADC tissues by TCGA, GEO database and Zhengzhou University (ZZU) ADC cohort analyses. *DDX11* overexpression was significantly correlated with the OS rate. Furthermore, univariate and multivariate analyses indicated that *DDX11* expression might be an independent prognostic element in ADC. These results showed that *DDX11* could serve as a promising biomarker for prognostic prediction in ADC.

To illustrate the diagnostic power of *DDX11* in ADC, we conducted a ROC curve analysis, and the results showed that the diagnostic value of ROC curves was satisfactory. To obtain convincing evidence of *DDX11* diagnostic power, we identified the diagnostic power of *DDX11* for ADC by a meta-analysis of previous studies downloaded from GEO ADC datasets. Therefore, *DDX11* might be a reliable diagnostic marker for ADC.

We further investigated the underlying mechanism of *DDX11* in promoting ADC tumorigenesis. Bioinformatic analysis indicated that high *DDX11* expression was closely linked to DNA replication and the cell cycle G1-S phase transition. Numerous studies have confirmed that the cell cycle is a complex and strictly controlled process 27 that is frequently dysregulated in tumorigenesis, including ADC 28, 29. Furthermore, previous studies have reported that several proteins, such as *FGF*^30^, *ERBB3* 31 and *MFN2* 32, may influence lung cancer progression through cell cycle pathways, Consistently, studies by Bhattacharya C et al. have demonstrated a key role for *DDX11* in the proliferation and cell cycle progression of advanced melanoma. In addition, our present study found that *DDX11* expression was positively associated with *CCNA2*, *CCNB1*, *CCNC*, *CCND1*, *CCNE1*, *CDK2*, *CDK4* and *CDK6*, which are involved in the cell cycle and DNA replication 33-36. These results suggested that *DDX11* might play a significant role in regulating the cell cycle G1-S phase transition and DNA replication in ADC progression.

## Conclusion

Our findings provide the first evidence that *DDX11* is overexpressed in ADC and has a close correlation with cancer progression, and the performance of *DDX11* in predicting a poor prognosis in ADC is also satisfactory. Taken together, these findings suggest that *DDX11* might be a potential prognostic and diagnostic biomarker for patients with ADC.

## Supplementary Material

Supplementary table.

## Figures and Tables

**Table 1:** The relationship between *DDX11* expression and the clinicopathological features of lung adenocarcinoma patients

Clinicopathological features		No. of cases (%)	*DDX11* expression level		*P value*
			Low	High	
Age (years)	≤40	41	15	26	0.458
	>40	45	20	25	
Gender	Female	40	18	22	0.448
	Male	46	17	29	
TNM	Ⅰ and Ⅱ	40	22	18	0.013*
	Ⅲ and Ⅳ	31	8	23	
	NA	15	5	10	
Histological type	Mixed Subtype	57	24	23	0.264
	Other Subtype	29	11	18	
Tumour size	≤3 cm	32	18	14	0.023*
	>3 cm	54	17	37	

**Notes:** *P<0.05. **P<0.01. **Abbreviations:** TNM: tumour-node-metastasis; NA: not available; HR: hazard ratio; CI: confidential interval; *DDX11*: DEAD/H-box helicase 11.

**Table 2:** Univariate and multivariate analyses of the overall survival of lung adenocarcinoma patients

Clinicopathological features		Univariate analyses			Multivariate analyses		
		HR	95% (CI)	P value	HR	95% (CI)	P value
Age (years)	≤40	1.000	0.919-1.710	0.547			
	>40	1.253					
Gender	Female	1.000	0.825-1.536	0.457			
	Male	1.125					
TNM stage	Stage I - II	1.000	1.577-3.076	<0.001**	1.000	1.546-3.021	<0.001**
	Stage III - IV	3.591			2.161		
Histological type	Mixed Subtype	1.000	0.700-1.585	0.262			
	Other Subtype	1.053					
Tumour size	≤3 cm	1.000	1.692-3.181	0.017*	1.000	1.246-1.729	0.175
	>3 cm	2.458			1.685		
*DDX11* expression	Low	1.000	1.783-2.983	0.024*	1.000	1.536-2.966	0.036*
	High	2.334			2.021		

**Notes:** *P<0.05. **P<0.01. **Abbreviations:** TNM: tumour-node-metastasis; HR: hazard ratio; CI: confidential interval; *DDX11*: DEAD/H-box helicase 11.
