# Supplementary material for: The Role of Upregulated DDX11 as A Potential Prognostic and Diagnostic Biomarker in Lung Adenocarcinoma
Source: J Cancer. 2019 Jul 10;10(18):4208–16. doi: 10.7150/jca.33457 (PMC6691710; doi:10.7150/jca.33457)
Supplement: Supplementary file 1 — Supplementary table. [file jcav10p4208s1.pdf]

Table S1 Characteristics of the GEO datasets.

**Cohort ID**	**Publication year**	**Country**	**RNAseq platforms**	**Total**	**Normal**	**Lung adenocarcinoma**	**Other**
GSE27262	2013	Taiwan, China	Affymetrix Human Genome U133 Plus 2.0 Array	50	25	25	0
GSE30219	2013	France	Affymetrix Human Genome U133 Plus 2.0 Array	307	14	85	208
GSE31210	2011	Japan	Affymetrix Human Genome U133 Plus 2.0 Array	246	20	226	0
GSE33532	2014	Germany	Affymetrix Human Genome U133 Plus 2.0 Array	100	20	40	40
GSE43767	2014	China	Whole Human Genome Microarray 4x44K G4112F	113	44	69	0
GSE7670	2007	Taiwan, China	Affymetrix Human Genome U133A Array	60	28	28	4
GSE10072	2008	USA	Affymetrix Human Genome U133A Array	107	49	58	0
